# Comparison of different load-velocity profiles and electromyography activity in the free-weight jump squat

**DOI:** 10.5114/biolsport.2026.159566

**Published:** 2026-04-20

**Authors:** Yi-Chien Chiang, Chieh-Ying Chiang, Jonathon Weakley

**Affiliations:** 1 Graduate Institute of Athletics and Coaching Science, National Taiwan Sport University, Taiwan; 2 Department of Sports Training Science-Combats, National Taiwan Sport University, Taiwan; 3 Taiwan Institute of Sports Science, Taiwan; 4 Sports Performance, Recovery, Injury and New Technologies (SPRINT) Research Centre, Australian Catholic University, Brisbane, QLD, Australia; 5 School of Behavioural and Health Sciences, Australian Catholic University, Brisbane, QLD, Australia; 6 Carnegie Applied Rugby Research (CARR) Centre, Carnegie School of Sport, Leeds Beckett University, Leeds, UK; 7 National Rugby Training Centre, Rugby Australia, Brisbane, Queensland, Australia

**Keywords:** Velocity-based training, Electromyography, Autoregulation, Ballistic exercise, Countermovement jump

## Abstract

Load-velocity profile (LVP) is an autoregulatory method in resistance training, yet the optimal LVP model for the free-weight jump squat (JS) remains unclear. This study therefore compared the accuracy of different LVP models and characterized their associated neuromuscular responses during the free-weight JS. In a cross-sectional, repeated-measures design, fifteen resistance-trained men first established their one-repetition maximum (1RM), and then performed free-weight JS at seven relative loads (20–80% 1RM). We compared generalized versus individualized, and linear versus polynomial LVP models. In addition, we measured mechanical outputs including peak and mean velocity, peak and mean power, and concentric phase time. Peak and mean neuromuscular activation of the vastus lateralis (VL) and medialis (VM) was measured using surface electromyography during the concentric phase. Individualized linear LVPs (*R*^2^ = 0.98–0.99) demonstrated superior accuracy and less prediction error compared to generalized models (*R*^2^ = 0.76–0.90), while polynomial models offered no advantage over linear models (p = 0.49–0.50). Peak and mean power was highest at 20% and 30% 1RM, respectively. Peak and mean muscle activation of the VL both peaked at 30% 1RM and decreased with increasing load. Practitioners should use individualized LVPs for accurate load prescription in the free-weight JS. Also, loads around 20–30% 1RM can induce high levels of acute mechanical power and VL muscle activation.

## INTRODUCTION

Ballistic training is often used for the development of neuromuscular recruitment and adaptations in muscular power [1, 2]. Its advantage in developing these qualities stems from its ability to elicit higher mechanical outputs compared to traditional non-ballistic exercises [3, 4]. The jump squat (JS) is a ballistic exercise frequently programmed to enhance lower-body power production [2, 3, 5]. Traditionally, JS exercise prescription has relied on using percentages of body mass (%BM) or one-repetition maximum half squat (%1RMHS) [5, 6]. However, using %BM or %1RM using %BM or %1RM may not account for daily fluctuations in strength, potentially leading to suboptimal training stimuli that could hinder adaptation [7]. Velocity-based training (VBT) serves as an alternative to percentage-based prescription and uses technology like linear position transducers (LPTs) to objectively measure movement velocity during exercise and provide real-time feedback [8]. This velocity data can then be used to construct load-velocity profiles (LVP), which can be used for 1RM estimation or load prescription [8–11].

Load-velocity profiles are one of the load autoregulation methods in resistance training [10], and can be fitted using either generalized (group-based) or individualized data. Given the high between-individual variability in ballistic movements [11], a direct comparison between different LVPs is essential for determining how to accurately prescribe load. Crucially, much of the existing research on LVPs in the JS has been conducted using Smith machines [12, 13], with Loturco et al. [12] finding LVPs a reliable method in the JS. On the other hand, free-weight JS may present higher ecological validity as they are more commonly used in athletic settings [14]. Free-weight exercises are often preferred in athletic programming as they have been shown to elicit greater muscle recruitment and acute hormonal responses, such as increased testosterone, compared to machinebased alternatives [15, 16]. Despite the benefits of such exercise, a direct comparison of different LVP models has not been performed in the free-weight JS. This leaves a significant knowledge gap for both researchers and practitioners, making it vital to investigate the comparative performance of LVP models to optimize load prescription.

While LVP provides a framework for prescribing external load, it does not characterize the underlying neuromuscular demands of those prescriptions. Integrating surface electromyography (sEMG) alongside LVP identifies the specific loads that maximize both mechanical outputs and muscle activation, ensuring the prescribed velocities align with intended motor unit recruitment [17]. For example, heavier loads in the back squat generate greater force production, typically reflected by a change in EMG activity and altered kinematics, such as reduced velocity [18]. Furthermore, while previous research has examined neuromuscular responses during the JS [19], they have focused primarily on integrated EMG amplitudes and were limited to a narrow range of loads. A more detailed analysis is needed to characterize root mean squared (RMS)-based EMG amplitudes [20], which are more representative of muscle activation strength during dynamic movements. Thus, this study aims to evaluate different LVP models (generalized vs. individualized; linear vs. polynomial) for goodness-of-fit and variability in the free-weight JS. Additionally, it characterizes the mechanical and neuromuscular responses across a range of relative loads (20–80% 1RM). We hypothesized that: (1) individualized LVPs would provide a more accurate fit than generalized profiles; (2) peak mechanical power and peak muscle activation of the quadriceps would be maximized at lighter relative loads (e.g., 20–40% 1RM).

## MATERIALS AND METHODS

### Design

This study was conducted on two separate days. In the first session, following a standardized warm-up and familiarization session, participants performed a 1RM test to assess maximal strength. In the second session, participants completed a progressive loading test (PLT) with the free-weight JS, where the load was increased from 20–80% 1RM-HS with 10% increments. Mechanical and electromyography data were continuously collected during PLT. The two sessions were separated by 24 to 48 hours.

### Sample size estimation and justification

The sample size was estimated *a priori* to ensure sufficient statistical power (0.80) for a null hypothesis significance test. The calculation was based on the primary analysis of this study, which is to determine if the correlation between relative load (%1RM) and barbell mean velocity was significantly different from zero. The statistical test used for power calculation was an exact test for a bivariate correlation coefficient. Based on prior LVP literature [9, 12, 13], a strong load-velocity relationship was expected. We conservatively powered the study to detect a correlation of *r* = 0.7. While prior literature [9, 12, 13] often reports higher correlation values, this lower threshold was selected *a priori* to ensure the study remained sufficiently powered even if there was a weaker load-velocity relationship. The calculation was performed using G*Power (v 3.1.9.7) with the following inputs: Test family = Exact, Statistical test = Correlation (Bivariate normal model), Tails = Two, Correlation (H1) = 0.70, *α* = 0.05, Power = 0.80, and Correlation (H0) = 0. This calculation resulted in a minimum required sample size of 13 participants. To account for a potential 15% dropout rate, the required sample size was increased to 15 participants.

### Participants

In this study, 15 healthy men (age = 23.9 ± 3.2 years; height = 175.7 ± 6.0 cm; body mass = 77.2 ± 9.0 kg; 1RM-HS = 181.0 ± 35.0 kg) with over six months of resistance training experience were recruited as participants. All participants were instructed to avoid any strenuous physical activity 48 hours prior to participation. All participants possessed 1RM-HS strength that was at least 1.5 times their body mass. Additionally, all participants were free from any acute lower-body injuries. This study was approved by the Institutional Review Board (IRB) at National Taiwan Sport University and written informed consent was obtained from the participants before study participation (approval code: NTSUIRB-113-088).

### Methodology

#### Familiarization

The familiarization session was held prior to commencing the experimental procedures. Participants were introduced to the commercial LPT system (GymAware [Model v5.2]; Kinetic Performance Technologies, Canberra, Australia), including the proper placement and attachment of the device. Practice jumps were performed with the LPT strapped onto the left side of the barbell with the attachment on the inside of the collar. Participants were all given instant velocity feedback both verbally and visually during exercise. This process was used in a bid to minimize learning effects and ensure data quality during subsequent testing sessions.

#### Half Squat One-Repetition-Maximum Test

A standardized warm-up was conducted, consisting of five minutes of aerobic exercise on a stationary bike, followed by five minutes of dynamic stretching consisting of leg swings, inchworms, walking lunges, lateral squats, and bodyweight squats. The 1RM testing protocol in this study has previously been detailed [21]. In short, the testing started from a warm-up set of eight repetitions at 50% of their estimated 1RM, followed by a set of five repetitions at 70% of their estimated 1RM, with three minutes of rest between warm-up sets. After the warm-up and an additional three-minute rest, participants performed single repetitions with progressively heavier weights, aiming to reach their 1RM within five attempts. The heaviest weight successfully lifted was recorded as the participant’s 1RM-HS.

The criterion of the correct lift was to squat down until a 90-degree knee angle. To ensure the reproducibility of the angle, an adjustable tripod with a photocell (Microgate, Bolzano, Italy) was placed on the right side of the bar. The photocell signals a sound when the bar crosses the depth linked to the 90° knee angle for each participant. Throughout the 1RM test, an experienced spotter was present to ensure participant safety, while all participants were provided with lifting belts during the testing.

#### Progressive Loading Test

The PLT was separated from the 1RM test by 24 to 48 hours to ensure full recovery from the maximal 1RM test and minimize any residual fatigue [11]. It began with the same standardized warm-up as the 1RM test, including five minutes of aerobic exercise on a stationary bike, followed by five minutes of dynamic stretching, with an addition of three warm-up sets of three repetitions of JS at 20%, 25%, and 30% of their 1RM-HS. The testing session started at their 20% 1RM-HS and increased in 10% increments up to 80% 1RM-HS using a fixed progressive loading order, which is consistent with load-velocity profiling literature [9, 12, 13]. To minimize potential fatigue or order effects, participants performed only two repetitions per load, with a 3-minute rest interval between loads. During exercise, the barbell was required to be in constant contact with participants’ upper back. The criterion of a correct lift was to squat down to a 90-degree knee angle, using the same experimental setup as in the 1RM testing. For each repetition, participants received real-time verbal and visual feedback of the velocity [22]. It was instructed that maximal effort was to be made in each repetition. Spotters and lifting belts were provided to standardize testing conditions and reflect common practice in resistance training to enhance safety. Throughout this test, electromyographic and mechanical data was continuously monitored.

#### Mechanical Data

The GymAware LPT was used to collect mechanical barbell data during the progressive loading test. This LPT employs a variable rate sampling technique with level crossing detection to optimize data interpretation. It then down-samples the data to 50 samples per second (50 Hz), ensuring noise is minimized when compared with highfrequency sampling [23]. The information was then transmitted via Bluetooth to a tablet for data collection. The LPT’s retractable cord was consistently placed at the left side of the barbell. This LPT has shown acceptable validity and reliability for collecting concentric phase mechanical variables [24, 25]. Concentric phase kinetic and kinematic outputs, including peak velocity (PV), mean velocity (MV), peak power (PP) and mean power (MP) and phase time (Con-Time) were collected in this study (Table 1). In the progressive loading test, the repetition with the fastest PV was selected for each load for analysis. This selection criterion was adapted from a similar study [26], and to ensure that the data reflects the participant’s maximal neuromuscular intent during exercise.

**TABLE 1:** Descriptive data including mechanical outputs and muscle activation across relative load ranges in the free-weight jump squat

Variables	Relative Load (% 1RM)						

	20	30	40	50	60	70	80
**Mechanical Outputs**							
PV (m · s^−1^)	2.62 ± 0.24	2.40 ± 0.22	2.17 ± 0.21	2.00 ± 0.20	1.81 ± 0.23	1.64 ± 0.22	1.46 ± 0.24
MV (m · s^−1^)	1.48 ± 0.14	1.31 ± 0.16	1.15 ± 0.13	1.06 ± 0.13	0.91 ± 0.12	0.79 ± 0.11	0.67 ± 0.14
PP (W)	5009 ± 1056	5260 ± 1186	5182 ± 1096	5115 ± 1090	5083 ± 1176	4837 ± 1132	4535 ± 1435
MP (W)	2755 ± 502	2680 ± 498	2452 ± 453	2384 ± 451	2206 ± 486	2031 ± 453	1743 ± 432
Con-Time (s)	0.52 ± 0.03	0.53 ± 0.04	0.54 ± 0.06	0.54 ± 0.07	0.55 ± 0.09	0.58 ± 0.10	0.65 ± 0.16

**Muscle Activation**							
pEMG-VL (%)	100.00 ± 0.00	128.08 ± 47.81	119.58 ± 43.50	94.99 ± 30.76	105.61 ± 37.62	98.50 ± 29.20	101.71 ± 40.98
pEMG-VM (%)	100.00 ± 0.00	112.35 ± 31.14	101.67 ± 40.55	92.26 ± 41.08	90.12 ± 34.98	94.95 ± 51.96	95.19 ± 61.31
mEMG-VL (%)	100.00 ± 0.00	114.93 ± 41.82	104.76 ± 33.65	87.16 ± 22.55	91.76 ± 30.42	83.84 ± 20.64	79.13 ± 26.54
mEMG-VM (%)	100.00 ± 0.00	104.50 ± 19.65	100.27 ± 35.12	87.73 ± 26.97	87.41 ± 32.35	84.82 ± 42.37	81.11 ± 53.44
iEMG-VL (%)	100.00 ± 0.00	119.96 ± 22.34	123.38 ± 22.75	124.80 ± 18.61	139.32 ± 27.79	145.31 ± 25.58	164.48 ± 43.21
iEMG-VM (%)	100.00 ± 0.00	116.94 ± 17.35	122.13 ± 24.09	126.90 ± 20.70	137.30 ± 28.62	144.29 ± 37.87	164.40 ± 56.24

Note. 1RM = one-repetition maximum; PV, peak velocity; MV, mean velocity; PP, peak power; MP, mean power; Con-Time, Concentric phase time; pEMG, peak electromyography value; mEMG, mean electromyography value; iEMG, integrated electromyography value; VL, vastus lateralis; VM, vastus medialis.

#### Electromyographic Measurements

Surface electromyography (sEMG) was used to record muscle activation during the progressive loading test. Prior to electrode placement, the skin overlying the target muscles was shaved and meticulously cleaned with alcohol wipes. Surface electrodes (Ag-AgCl, 4.0 × 3.2 cm; 3M Health Care, Ontario, Canada) were placed on the target muscle’s belly according to SENIAM (Surface EMG for Non-Invasive Assessment of Muscles) recommendations [27] on the vastus lateralis (VL) and vastus medialis (VM) of the right leg. The reference electrode was placed on the patella of the same limb. The electrodes and wires were secured with adhesive tape (3M, Canada), with an inter-electrode distance of 20 mm. The sEMG signals from each electrode were amplified (× 1000) and sampled at 1000 Hz. Electrodes were connected to an amplifier (BioNomadix 2Ch Wireless EMG Amplifier, Biopac System, Goleta, CA) and streamed continuously through an analog to digital converter (MP160, Biopac System, Goleta, CA).

Vertical ground reaction force (vGRF) was also recorded by a set of force plates recording at 1000 Hz (Type 2812A, Kistler, Winterthur, Switzerland) and time-synchronized with the EMG signal. Acceleration was calculated from the vGRF data, and velocity was subsequently derived using the trapezoidal rule integration. Velocity data were then used to identify the concentric phase of each repetition [28].

Electromyography signals were filtered with a fifth-order Butterworth digital filter (10–500 Hz) and rectified before analysis. The root mean square (RMS) window was used to smooth the rectified signal using a 100-ms moving window. Following established methods [29, 30], the formula applied was:

$$RMS=\sqrt{\frac{\Sigma_{n=1}^{N}X_{n}^{2}}{N}}$$

where *X*_n_ is the value of the rectified sEMG signal at sample point *n*, and *N* is the sample size within the moving window. Analysis of the sEMG data was performed to the identified concentric phase of each repetition. Amplitude parameters were derived from the processed signal. Mean electromyography (mEMG) and peak electromyography (pEMG) were calculated from the smoothed (RMS) signal within the concentric phase. Integrated electromyography (iEMG) was calculated as the integral of the rectified, non-windowed sEMG signal. The values of each EMG parameter (mEMG, pEMG, iEMG) were normalized to the first set of the progressive loading test (i.e., 20% 1RM-HS) for each participant. This dynamic normalization strategy was selected over the traditional isometric maximal voluntary contraction (MVC) method because it accounts for task-specific motor unit recruitment, while also maintaining the same level of test-retest reliability. [30, 31]. This ensures that changes in EMG amplitude accurately reflect the neuromuscular activation within the mechanical constraints of the jump squat.

### Statistical Analyses

Normal distribution of the data was checked using Shapiro-Wilk test before analysis. Statistical analyses were performed in RStudio (Posit, Boston, MA) using the R programming language (Version 4.4.2, R Foundation, Vienna, Austria). Descriptive data were presented as mean ± SD. The level of significance was set at α = 0.05 for relevant tests. The relationship between relative load (%1RM) and velocity (PV, MV) was established by fitting linear and second-order polynomial models to the generalized and individualized data using the *lm* function. The goodness of fit of the linear regressions were assessed by correlation coefficient (*r*), coefficient of determination (*R*^2^), and the root mean square error (RMSE). Fisher *r* to *z* transformations were used to determine significant differences between linear and second-order polynomial model performance. Between-participant variability at each relative load was assessed using the coefficient of variation.

$$CV[\%]=\frac{\text{Between}-\text{participant SD}}{Mean}\times 100$$

To examine the effects of load on mechanical outcomes and muscle activation, linear mixed effects models were built using the *lmer* function from the *LmerTest* package. A model was built for each outcome variable of interest, and a clustered structure with participants nested within each relative load. Within each model, relative load was used as a fixed effect, while random intercepts were fit for each participant. The distribution of the residuals was checked to see whether they follow a normal distribution. Post-hoc comparisons were adjusted using Tukey’s HSD to control the family-wise error rate within each mixed-model family, using the *emmeans* package. To interpret the magnitude of these differences, effect sizes with Hedge’s correction and 95% confidence intervals were estimated from each model using the *t_to_d* function from the *effectsize* package. Effect sizes were interpreted as: 0.0–0.19, trivial; 0.2–0.59, small; 0.6–1.19, moderate; 1.2–1.99, large; 2.0–3.99, very large; and > 4.0, extremely large [32].

Within-session reliability was assessed for all mechanical and electromyographic variables using the two repetitions performed at each load, while quantified using Intraclass Correlation Coefficients (ICC, model [3, 1]). Variables demonstrated ICC values of 0.82 for PP, 0.93 for PV, 0.82 for MP, 0.95 for MV, 0.85 for mEMG-VL, 0.83 for mEMG-VM, 0.69 for pEMG-VL, 0.76 for pEMG-VM, 0.84 for iEMG-VL, and 0.90 for iEMG-VM.

## RESULTS

### Model Performance of the Load-Velocity Profiles

**FIG. 1:**
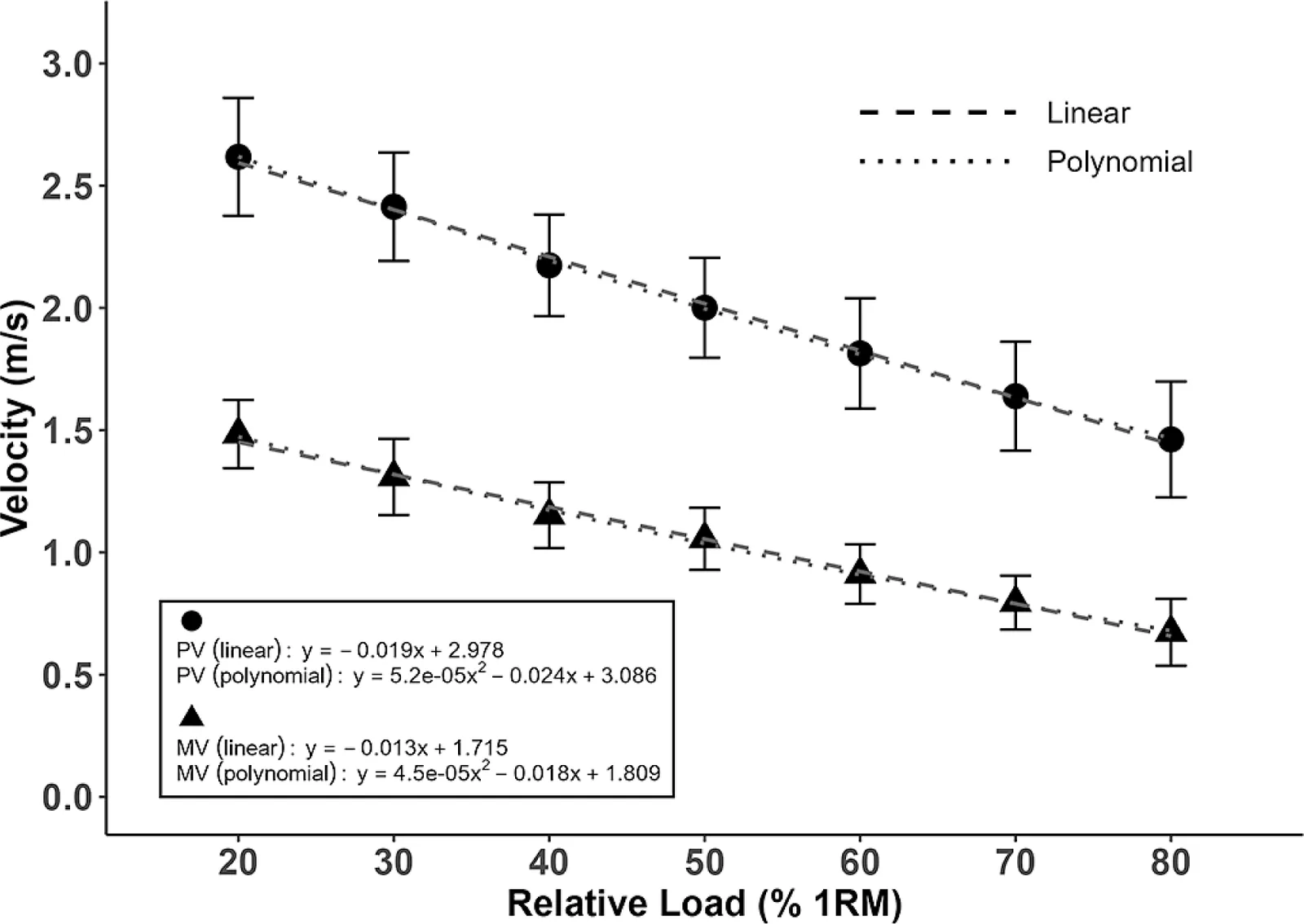
Group mean (SD) values from linear and polynomial load-velocity profiles for the free-weight jump squat. Linear regression (---) and second-order polynomial (···) are presented with respective regression equations in the box located at the bottom-left. 1RM = one-repetition maximum; PV, peak velocity; MV, mean velocity.

**FIG. 2:**
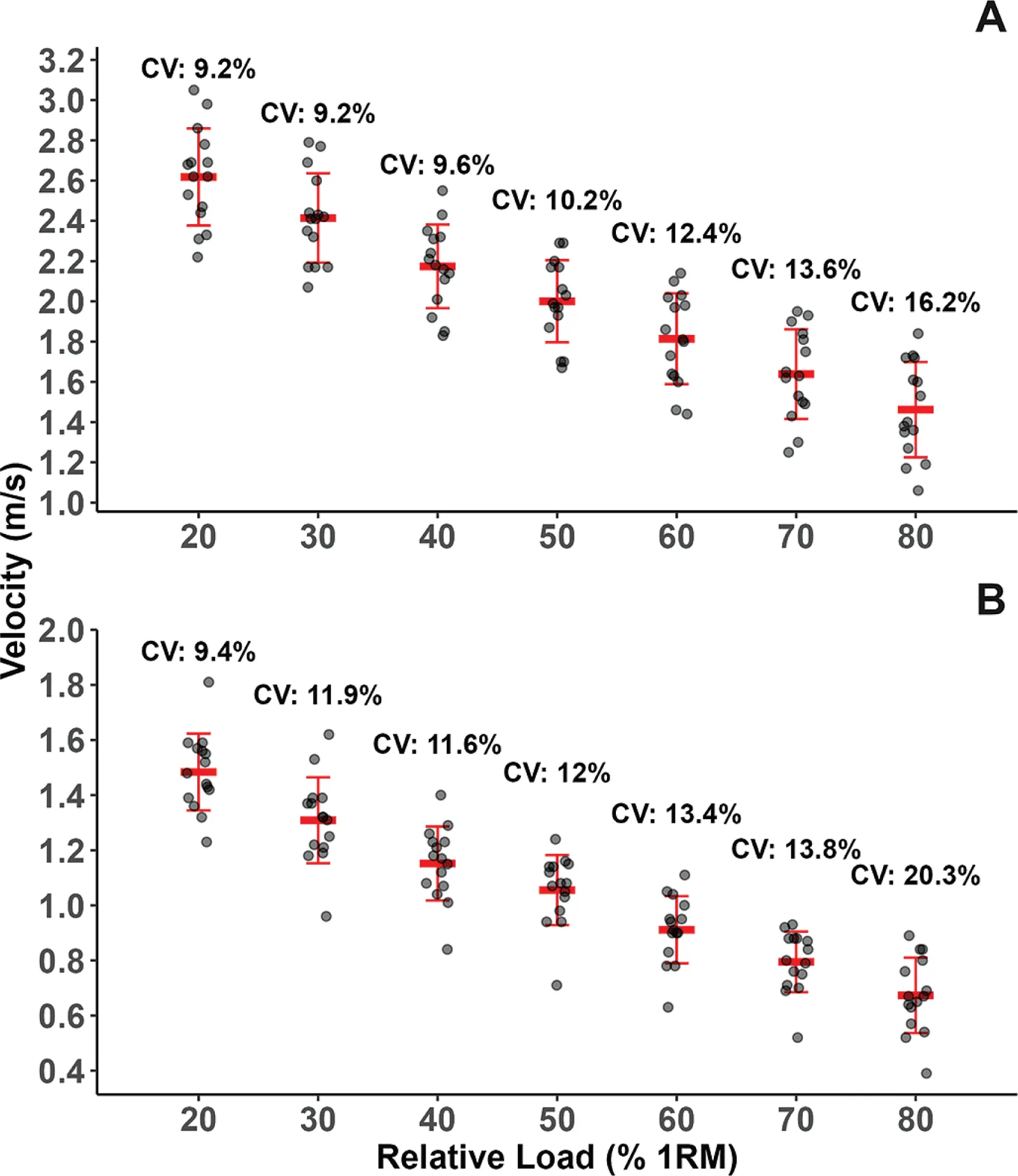
Between-participant variability for (A) PV and (B) MV for the free-weight jump squat. Individual datapoints are illustrated as filled circles. Group mean (SD) are represented by the red horizontal bar (error bars). CV values are displayed above each percentage load. 1RM = 1-repetition maximum; CV = coefficient of variation; PV = peak velocity; MV = mean velocity.

The relationships between relative load (% 1RM) and both PV and MV were examined using linear and second-order polynomial regression models. As illustrated in Figure 1, both PV and MV demonstrated a significant inverse relationship with relative load (*p* < 0.001). For generalized data, both linear and second-order polynomial models indicated a very strong relationship for PV and even stronger correlations for MV (Table 2). No significant differences were present between the linear and second-order polynomial model coefficients (PV: *p* = 0.50; MV: *p* = 0.49). When examining individualized data, the relationships were stronger for all models but especially stronger for PV (Table 2). Between-participant variability for PV and MV across the different relative loads is presented in Figure 2.

**TABLE 2:** Linear regression and second-order polynomial model performance outcomes for the free-weight jump squat, with generalized and individualized data

	Linear Regression						Second-Order Polynomial					

	Generalized			Individualized			Generalized			Individualized		

Response	*r*	*R* ^2^	RMSE (m · s^−1^)	*r*	*R* ^2^	RMSE (m · s^−1^)	*r*	*R* ^2^	RMSE (m · s^−1^)	*r*	*R* ^2^	RMSE (m · s^−1^)
PV	0.87	0.76	0.22	0.99	0.98–0.99	0.03–0.06	0.87	0.76	0.22	0.99	0.98–0.99	0.01–0.05

MV	0.90	0.81	0.13	0.97–0.99	0.94–0.99	0.03–0.06	0.90	0.81	0.13	0.98–0.99	0.95–0.99	0.01–0.06

Note. PV = peak velocity; MV = mean velocity; RMSE = root mean square error.

### Effect of Load on Mechanical Data

Significant main effects of relative load were found on PP (*p* < 0.001), MP (*p* < 0.001), and Con-Time (*p* < 0.001).

Post-hoc analyses for PP indicated that changes did not reach significance across 20–60% 1RM, with 30% 1RM having the highest PP. A significant moderate decrease in PP was observed at 80% 1RM when compared to 20% 1RM (*g* = -0.79, *p* = 0.009), and at 70% 1RM when compared to 30% 1RM (*g* = -0.79, *p* = 0.028).

For MP, a progressive decrease was observed with increasing relative load. Significant small to extremely large decreases were evident when comparing 40%, 50%, 60%, 70% and 80% 1RM to 20% 1RM (*g* = -1.23 to -4.11, all *p* < 0.001).

Con-Time generally demonstrated an increasing trend with greater relative loads. A significant large increase was observed at 80% 1RM when compared to 20% 1RM (*g* = 1.36, *p* < 0.001), and a significant moderate increase was noted at 70% 1RM compared to 20% 1RM (*g* = 0.66, *p* = 0.050).

### Effect of Load on Electromyography Measures

Significant main effects of relative load were found on pEMG (*p* = 0.012), mEMG (*p* < 0.001), and iEMG for the VL (*p* < 0.001). For pEMG-VL, the highest value was observed at 30% 1RM which has a significant moderate increase over 50% 1RM (*g* = -0.71, *p* = 0.025). For mEMG-VL, the highest value was also observed at 30% 1RM, and significant moderate differences were present when comparing 50% 1RM (*g* = -0.76, *p* = 0.013), 70% 1RM (*g* = -0.85, *p* = 0.003), and 80% 1RM (*g* = -0.98, *p* < 0.001) to the 30% 1RM load. In contrast, iEMG-VL demonstrated an increasing trend as the load became heavier. The magnitude of EMG differences between relative load is illustrated in Figure 4.

**FIG. 3:**
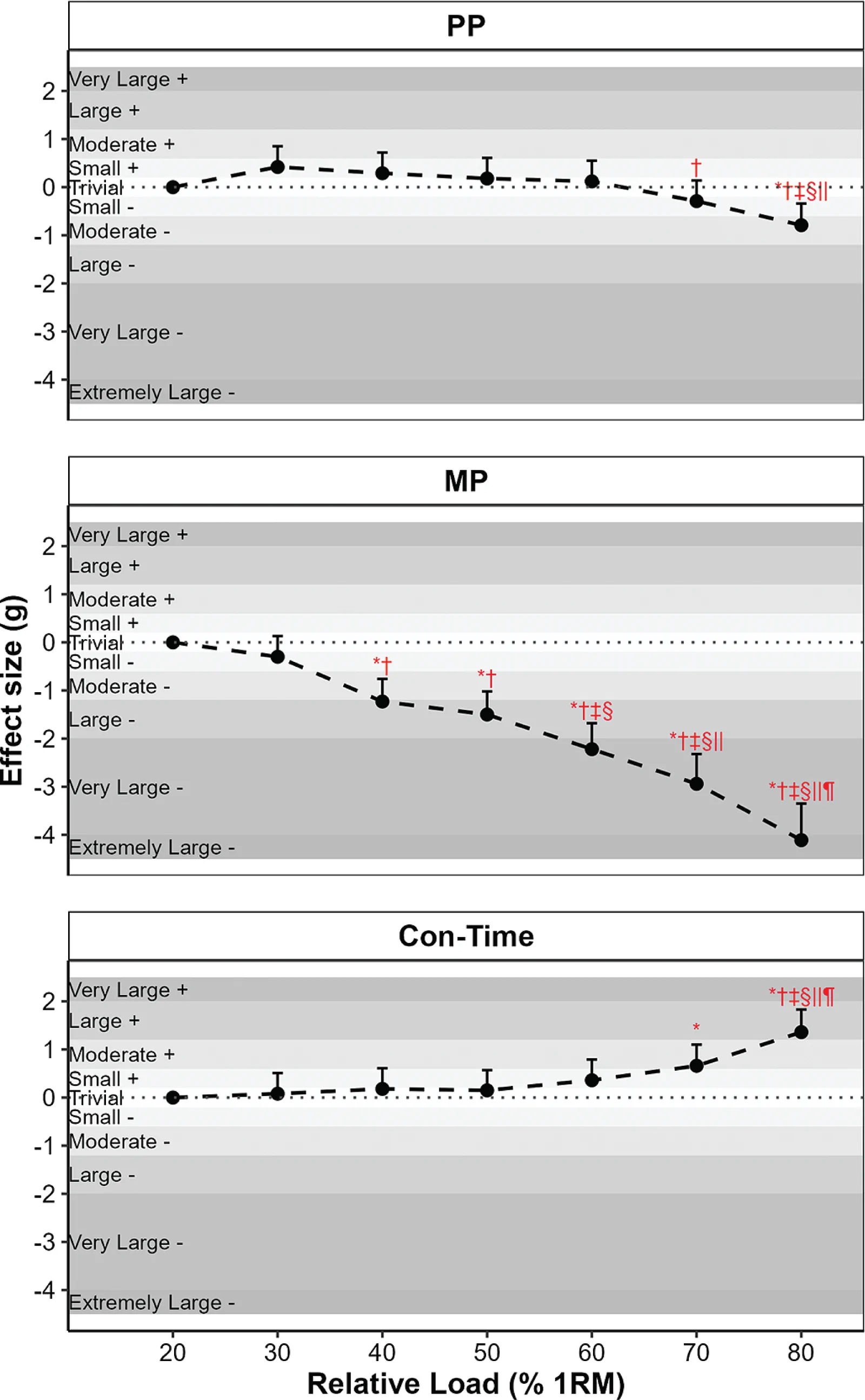
Effect sizes (Hedges’ g) for mechanical variables across relative loads (% 1RM) during the free-weight jump squat. Data points represent the mean effect size, and error bars indicate the 95% confidence intervals. The shaded areas represent interpretations of effect size magnitudes. Significant differences from the 20%, 30%, 40%, 50%, 60%, 70% 1RM condition are indicated by symbols (respectively, *, †, ‡, §, ||, ¶). 1RM = 1-repetition maximum; PP, peak power; MP, mean power; Con-Time, Concentric phase time.

**FIG. 4:**
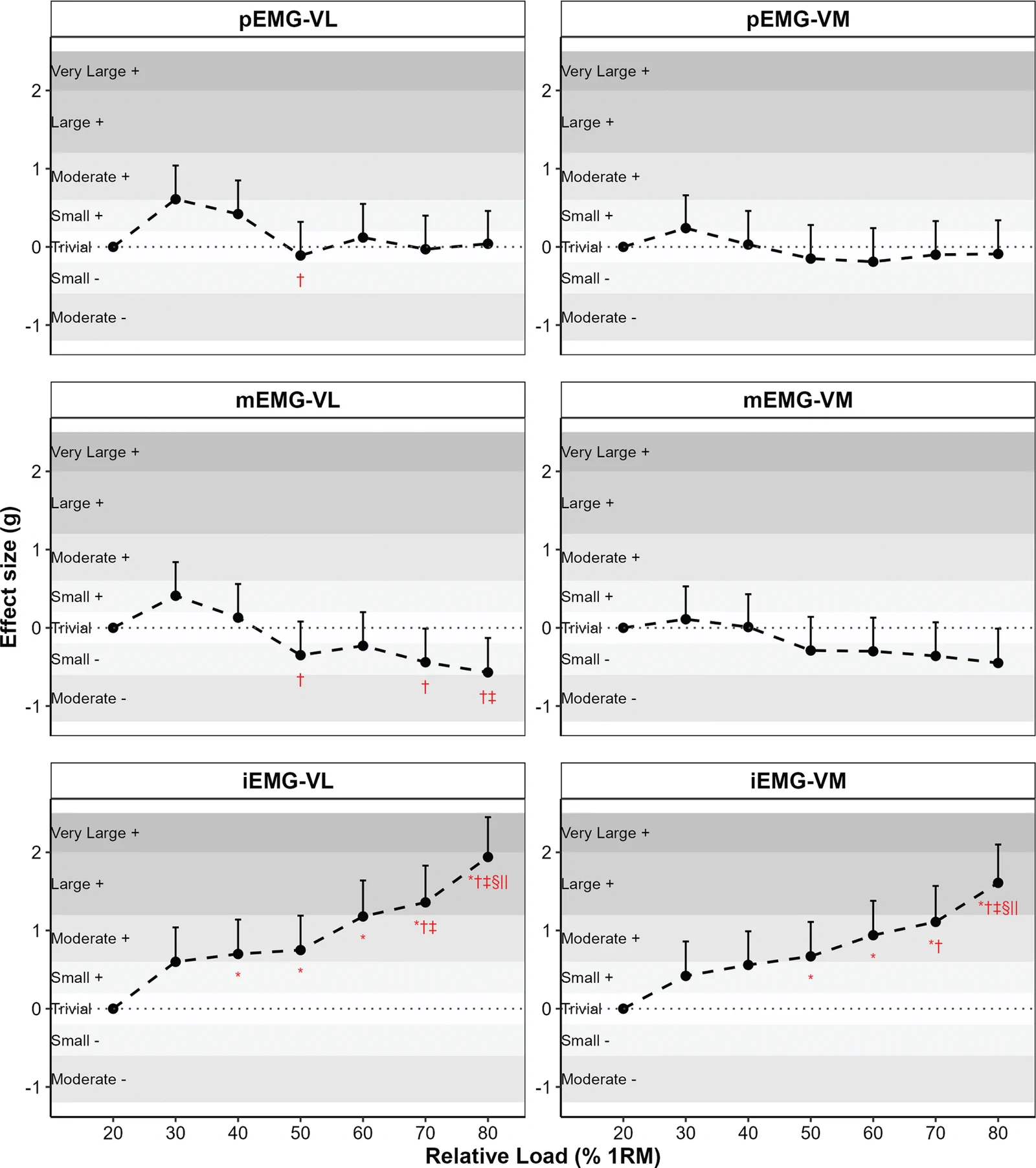
Effect sizes (Hedges’ g) for electromyography (EMG) variables of the vastus lateralis (VL) and vastus medialis (VM) across relative loads (% 1RM) during the free-weight jump squat. Data points represent the mean effect size, and error bars indicate the 95% confidence intervals. The shaded areas represent interpretations of effect size magnitudes. Significant differences from the 20%, 30%, 40%, 50%, 60%, 70% 1RM condition are indicated by symbols (respectively, *, †, ‡, §, ||, ¶). 1RM = 1-repetition maximum; pEMG, peak electromyography value; mEMG, mean electromyography value; iEMG, integrated electromyography value; VL, vastus lateralis; VM, vastus medialis.

For the VM, no significant main effect of load was found for pEMG (*p* = 0.531) or mEMG (*p* = 0.081), although a significant main effect was found for iEMG (*p* < 0.001). As illustrated in Figure 4, iEMG-VM demonstrated an increasing trend with heavier loads.

## DISCUSSION

This study aimed to evaluate different LVP models (generalized vs. individualized; linear vs. polynomial) for goodness-of-fit and interparticipant variability in the free-weight JS. Additionally, it characterized the mechanical and neuromuscular responses across a wide range of relative loads (20–80% 1RM). Our findings reveal individualized LVPs provide the most accurate and practical method for load prescription, while mechanical output and muscle activation patterns are distinctly modulated by relative load, with 20–30% 1RM being optimal intensities for achieving peak mechanical power output, which concurrently corresponded with high levels of muscle activation.

### Load-Velocity Profiles

We confirmed a strong inverse relationship between relative load (% 1RM) and movement velocity, both PV and MV, during the free-weight JS. This was evidenced by the generalized models yielding *R*^2^ of 0.76 for PV and 0.81 for MV (Table 2). This phenomenon, where increased muscle shortening velocity limits actin-myosin cross-bridge formation and thus force production, dictates that as external load increases, movement velocity inherently decreases [1]. Furthermore, the specific characteristics of the JS LVP observed herein suggest that these profiles are exercise-dependent, differing from those of traditional, non-ballistic squatting exercises [10, 11] due to the explosive, accelerative nature of the JS. The strong load-velocity relationships we found for the free-weight JS are somewhat different from those reported in the Smith machine JS. For instance, Pérez-Castilla et al. [13] established an LVP in the Smith machine JS with an *R*^2^ of 0.96 for mean propulsive velocity, and Loturco et al. [12] reported *R*^2^ values of 0.91 for PV and 0.92 for MV in the same exercise. In comparison, we discovered slightly lower *R*^2^ values (PV: 0.76, MV: 0.81) using the generalized model in the free-weight JS. The nature of the free-weight JS likely introduces greater movement variability than machine-based alternatives [14], potentially reducing the robustness of generalized models. This highlights the need for individualized LVPs as our results reflected higher predictive performance in these models.

A critical finding of this study was the considerable between-participant variability in velocity outputs at given relative loads, with CV progressively increasing across 20–80% of 1RM (Figure 2). This trend may be explained by the varying strength levels within our cohort (1RM-HS 181.0 ± 35.0 kg), different fiber type and recruitment patterns [11], or different training background of the subjects [12, 13]. This limits the ability for generalized predictive models to predict load. Consequently, individualized LVPs demonstrated markedly stronger relationships and lower prediction errors. The superior accuracy of individualized profiles is consistent with findings for other freeweight exercises [10, 11]. This is particularly relevant as PV is often the metric recommended for constructing LVPs in ballistic exercises [8]. These factors together emphasize the necessity of individual profiling for effective VBT program design.

Despite the potential for curvilinearity in load-velocity relationships, this study found no statistically significant differences in goodness-of-fit between linear and second-order polynomial regression models for describing the LVP of the free-weight JS (PV: *p* = 0.50; MV: *p* = 0.49). Practically, this suggests simpler linear models suffice for accurately profiling the load-velocity relationship in healthy recreationally trained males, offering an accessible method while maintaining predictive power. This aligns with prior research on other exercises, which also found minimal advantage in more complex polynomial models [10, 11].

### Mechanical Outputs and Muscle Activation Across Loads

Increasing external load during the free-weight JS altered mechanical outputs, consistent with similar studies [4, 12]. As load increased, movement velocities (both PV and MV) progressively decreased, while concentric phase time correspondingly increased. Mean power also consistently declined with heavier loads. Peak power remained relatively stable across lighter to moderate loads (20–60% 1RM) and peaked at around 30% 1RM, before significantly declining at heavier intensities (70–80% 1RM). This also aligns with previous findings [4], which suggests that lighter loads in ballistic exercises often elicited greater velocity and power outputs than moderate and heavy loads. However, our finding provides a comparison to Cormie et al. [33], who reported that peak power was maximized with no external load in untrained participants. The discrepancy likely reflects the superior relative strength of this study’s cohort (trained participants with 1RM-HS > 1.5x body mass) compared to the untrained subjects in prior research [33].

Electromyographic responses exhibited distinct load-dependent characteristics. As the JS is a quadriceps-dominant exercise, this study focused on the EMG activity of the VL and VM as key representatives of this muscle group [27]. Peak and mean activation levels of the VL were generally highest at 30% 1RM, tending to decrease at heavier loads, while the VM showed a similar, albeit non-significant trend for these parameters. This EMG response aligns with previous findings, which showed a slight but non-significant difference of activation in the two muscles during resistance exercises [30]. Notably, this study appears to be the first to comprehensively detail these specific load-EMG relationships in the free-weight JS.

The observed EMG responses in this study contrast with typical findings in non-ballistic exercises, such as the back squat, where muscle activation often demonstrates a more direct increase with load [18]. Our EMG findings build upon and expand the work of Nuzzo & McBride [19], whose research focused on iEMG over a narrower load range. By analyzing RMS-based amplitudes across a wider spectrum up to 80% 1RM, our results provide a more detailed characterization of muscle activation. We found that peak VL activation occurred around 30% 1RM, coinciding with the study’s highest mechanical peak power output (Figure 3). This suggests the 30% 1RM load represents an optimal balance of force and velocity demands to elicit maximal VL activation. Moreover, the reduction in movement velocity at heavier loads, coupled with the additional existence of sticking regions [34], also likely contributed to the decline in these EMG amplitude measures in heavier loads. Concurrently, iEMG for both VL and VM significantly increased with load, which can be attributed to the longer concentric phase durations required to lift heavier loads (Figure 3), resulting in greater cumulative muscle activity [35].

While this study is among the first to investigate different LVP models alongside electromyographic responses across a wide spectrum of relative loads in the free-weight JS, some limitations should be acknowledged. First, it should be noted that the load-velocity relationship may be specific to the strength characteristics of our participants (1RM-HS = 181.0 ± 35.0 kg). Practitioners intending to use LVPs should establish individualized profiles of their own athlete populations through regular testing. Second, it should be acknowledged that our choice to analyze only the fastest repetition per load prioritizes peak performance and may not fully represent the outputs an athlete might produce across multiple sets in a training session. Furthermore, the use of sEMG during explosive, dynamic contractions like the JS presents inherent complexities [31]. For example, electrode displacement relative to muscle fibers may alter due to rapid changes in joint angles and muscle length, which could have influenced the recorded sEMG amplitudes [30]. Additionally, we normalized sEMG amplitudes to a dynamic contraction value (i.e., the 20% 1RM load) rather than adopting the isometric MVC method [31]. This normalization approach reflects activation values relative to a submaximal condition, which may limit direct comparisons with existing literature where the MVC method was mostly adopted. Finally, while our sample size of 15 was appropriately powered for the primary LVP analysis, we acknowledge that it may limit the statistical power of the secondary analysis (i.e., comparisons of mechanical and EMG outcomes across seven loads). Future studies with larger samples may be required to further investigate these differences between-load.

This study’s findings offer several practical applications for optimizing free-weight JS training. Practitioners should prioritize individualized LVPs during the free-weight JS, due to their superior accuracy in predicting relative load, while noting that simple linear models suffice for prediction. As for mechanical and neuromuscular outcomes, this paper contribute to the previous findings [19] by providing a more comprehensive EMG analysis. Relative loads around 20–30% 1RM-HS can maximize power output and quadriceps muscle activation. By integrating these insights, individualized LVPs can be utilized to prescribe training loads aimed at achieving specific training stimulus. For instance, since the loads that maximize peak power (20–30% 1RM) also elicited high levels of muscle activation, practitioners can use individualized LVPs to target the corresponding velocity (e.g., PV around 2.40–2.62 m · s^−1^) to emphasize power-oriented training.

## CONCLUSIONS

This study compares the accuracy of different LVPs, and provides a detailed profile of acute muscle activation levels across loads in the free-weight JS. The results confirm a strong linear and inverse load-velocity relationship that is best characterized using individualized profiles. For comparison across loads, peak power output was maximized at approximately 30% of 1RM-HS, while mean power progressively decreased with increasing load. Peak and mean EMG amplitudes of the VL were generally higher at around 20–30% 1RM, whereas integrated amplitudes increased with heavier loads, though caution is advised when generalizing these effects to the entire quadriceps group. These findings provide practitioners with evidence and confidence to use LVP in the free-weight JS to target acute neuromuscular and mechanical responses. Future research should determine if these relationships remain consistent across different populations and training backgrounds, and if training at these identified loads translates to superior long-term performance adaptations.
